# *GSTM1* copy number variation in the context of single nucleotide polymorphisms in the human *GSTM* cluster

**DOI:** 10.1186/s13039-016-0241-0

**Published:** 2016-04-19

**Authors:** Andrey V. Khrunin, Irina N. Filippova, Aydar M. Aliev, Tat’yana V. Tupitsina, Petr A. Slominsky, Svetlana A. Limborska

**Affiliations:** Department of Molecular Bases of Human Genetics, Institute of Molecular Genetics of Russian Academy of Sciences, Kurchatov sq. 2, Moscow, 123182 Russia

**Keywords:** DNA polymorphism, *GSTM1* deletion, *GSTM1* copy number polymorphism, Single nucleotide polymorphism, Haplotype

## Abstract

**Background:**

*GSTM1* gene deletion is one of the most known copy number polymorphisms in human genome. It is most likely caused by homologous recombination between the repeats flanking the gene. However, taking into account that the deletion has no crucial effects on human well-being, and the ability of other *GSTMs* to compensate for the lack of *GSTM1*, a role for additional factors affecting *GSTM1* deletion can be proposed. Our goal was to explore the relationships between *GSTM1* deletion polymorphism and single nucleotide polymorphisms (SNPs) in the region of the *GSTM* cluster that includes *GSTM2*, *GSTM3*, *GSTM4*, and *GSTM5* in addition to *GSTM1*.

**Results:**

Real-time polymerase chain reaction was used to quantify the number of *GSTM1* copies. Fourteen SNPs from the region were tested and their allelic patterns were compared in groups of Russian individuals subdivided according to their *GSTM1* deletion genotypes. Linkage disequilibrium-based haplotype analysis showed substantial differences of haplotype frequencies between the groups, especially between individuals with homozygous *GSTM1* −/− and +/+ genotypes. Exploration of the results of phasing of *GSTM1* and SNP genotypes revealed unequal segregation of *GSTM1* + and − alleles at different haplotypes.

**Conclusions:**

The observed differences in haplotype patterns suggest the potential role of genetic context in *GSTM1* deletion frequency (appearance) and in the determination of the deletion-related effects.

## Background

Glutathione-*S*-transferases (GSTs) are a group of enzymes that play an important role in the metabolism and detoxification of a wide range of exo- and endogenous compounds. Functionally, GSTs act by conjugating electrophilic centers of the compounds with molecules of reduced glutathione. The reaction products are generally less reactive and readily excreted [1, 2]. Although many GST substrates can be potentially harmful to cellular macromolecules, some GST genes, particularly *GSTT1* and *GSTM1*, are homozygously deleted in a high percentage (15 to 60 %) of individuals in various human populations [3]. *GSTT1* and *GSTM1* copy number variations are most likely caused by homologous recombination between the repeats flanking the both genes [4, 5]. However, the reasons for the high frequency of the deletions across different human populations are not well understood, especially if considered in the context of their associations with a higher risk for the development of a variety of pathophysiological conditions in individuals carrying deletion phenotypes [6]. A possible explanation could be the capability of other GSTs to compensate, at least in part, for the absence of GSTT1- and GSTM1-related enzyme activities under normal conditions [1]. Recent studies [7, 8] showed distinct connections between the levels of GST enzymatic activity and the activity of genes belonging to the same class (i.e., *GSTT2B* and *GSTM2*), evidently suggesting a role for local genetic context in the deletion frequency (deletion appearance).

In the current study, we investigated relationships between *GSTM1* deletion polymorphism and SNP-based haplotypes in the region of the *GSTM* cluster that includes *GSTM2*, *GSTM3*, *GSTM4*, and *GSTM5* in addition to *GSTM1*. The obtained results demonstrated substantial differences in haplotype distribution between groups of individuals subdivided according to *GSTM1* deletion genotypes.

## Methods

Blood samples were obtained with informed consent from Russian donors from three locations in the European part of Russia (Andreapolsky District of the Tver Oblast, *n* = 96; Muromsky District of the Vladimir Oblast, *n* = 96; and Kursky and Oktyabrsky districts of the Kursk Oblast, *n* = 93). The ethnicity of the donors was determined by an interview. To be included, individuals had to be unrelated and represent the native ethnic group in the regions studied (i.e., they belonged to at least the third generation living in a particular geographic region). The interview protocol and informed-consent form were approved by the Ethic Commission of Institute of Molecular Genetics of the Russian Academy of Sciences.

The DNA was isolated from peripheral leukocytes of the blood with a standard technique using proteinase K treatment and phenol–chloroform extraction [9].

To subdivide the individuals according to *GSTM1* deletion polymorphism, the results of two genotyping methods were used. The first method was based on the simultaneous amplification of a site on *GSTM1* with a region of another gene used as an internal control. The method makes it possible to identify individuals with a homozygous deletion genotype (i.e., they have no *GSTM1* copies at all; *GSTM1* −/− genotype). This approach was used in our previous study, and data for individuals with a *GSTM1* −/− genotype were satisfactorily obtained [10]. Individuals with one or two copies of the *GSTM1* gene are not distinguished by this method. To differentiate between individuals with one (i.e., heterozygotes; *GSTM1* −/+) or two (i.e., normal homozygotes; *GSTM* +/+) copies of *GSTM1*, quantitative real-time PCR was used. It was conducted using a TaqMan (5′-nuclease) assay system with signal from a *GSTM1*-specific probe that was normalized to the signal from a reference autosomal β-2-microglobulin gene (*B2M*). Primers and probes used to amplify the *GSTM1* and *B2M* regions are presented in Table 1.

**Table 1:** Primers and probes used in the study

Gene	Sequence^a^
*B2M*	Forward primer: 5′-TTGTTTCACTGTCCTGAGGACTATTTAT-3′
	Reverse primer: 5′-ATGTTACTCTGTCAATGTTCTCCACAT-3'
	Probe: 5′-ROX-CTCTAACATGATAACCCTCAC-BHQ2-3′
*GSTM1*	Forward primer: 5'-CTGAGCCCTGCTCGGTTTAG-3'
	Reverse primer: 5'-ATGGGCATGGTGCTGGTT-3'
	Probe: 5'-FAM-CTGTCTGCGGAATC-BHQ1-3'

^a^sequences of primers and probes for *B2M* and *GSTM1*are taken from the studies Covault et al. [25] and Nørskov et al. [26], respectively

PCR was performed in 25 μL of 1× PCR buffer, containing 2.5 mM MgCl_2_, 200 μM each of dNTP, 20 pM each of *GSTM1* and *B2M* primers, 1.25 units of Hot-Rescue Taq DNA polymerase (Syntol, Moscow, Russia), 10 pM and 5 pM of *GSTM1*- and *B2M*-specific probes, respectively, and 10–20 ng of genomic DNA. Thermal cycling and fluorescence intensity measurement were conducted using a StepOnePlus Real-Time PCR System (Applied Biosystems, Waltham, MA, USA). The samples were initially incubated for 10 min at 95 °C and then cycled 35 times at 95 °C for 20 s, followed by 60 °C for 60 s. All samples were tested in pentaplicate.

To quantify the number of *GSTM1* copies a comparative Ct method was used [11]. The ratio (R) of the *GSTM1* to *B2M* gene dosage was calculated using the formula R = 2^−ΔΔCt^, where ΔΔCt = (Ct_control_B2M_ – Ct_control_GSTM1_) – (Ct_sample_B2M_ – Ct_sample_GSTM1_). We used a control sample known to be heterozygous for *GSTM1*. Based on the observed variability, R values higher than 1.4 were interpreted as an indication that the sample carried two functional variants (two copies) of *GSTM1*. Ratios between 0.7 and 1.3 were considered attributable to samples with heterozygous deletions (containing one *GSTM1* copy).

Population SNP genotypes were obtained from our previous study, in which they were generated using Illumina Human CNV370-Duo and Human 660 W-Quad chips [12]. The set of SNPs was chosen by considering the chromosomal region in which the genes of the *GSTM* family were located.

Data on individual *GSTM1* deletion genotypes and SNP genotypes in other populations (i.e., CEU) were obtained from the database of the 1000 Genomes Project [13].

To explore patterns of genetic variation across *GSTM* cluster, a haplotype analysis was performed. Two approaches were used. The first was based on an analysis of haplotypes in the haplotype blocks (haploblocks). The haplotype blocks were defined using a block definition based on the linkage disequilibrium (LD) measure *D*′ and its confidence interval. The corresponding pairwise LD statistics between SNPs and the frequencies of haplotypes were estimated using Haploview software (version 4.2) [14]. Comparisons of haplotype frequencies between groups of individuals were performed using GraphPad InStat (version 3.00, GraphPad Software, San Diego, CA, USA). *P* < 0.05 was considered significant.

The second approach consisted of exploring the patterns of haplotypes at which the GSTM1 null and non-null allele(s) segregated in populations. To obtain such structural haplotypes, the *GSTM1* and SNP genotypes of populations were phased together using the Beagle software package (version 4, release 1399) using default parameters [15]. Visualization of the sets of phased *GSTM1* and SNP alleles was conducted using a custom R script kindly provided by Robert E. Handsaker (Broad Institute of MIT and Harvard, Cambridge, MA, USA) [16].

## Results

In previous studies based on both whole-genome polymorphism analysis and testing SNPs in *GSTA* and *GSTM* clusters, high similarity between three Russian populations from the Central European part of Russia was demonstrated [12, 17]. To create a more effective sample, particularly in the context of generally under-represented *GSTM1* +/+ genotype carriers, three Russian populations were combined. In total, 128 individuals with a *GSTM1* −/− genotype, 121 individuals with a *GSTM1* −/+ genotype, and 36 individuals with a *GSTM1* +/+ genotype were detected in the sample. Genotype frequencies did not differ from those predicted by the Hardy–Weinberg rule (*P* = 0.45).

**Fig. 1:**
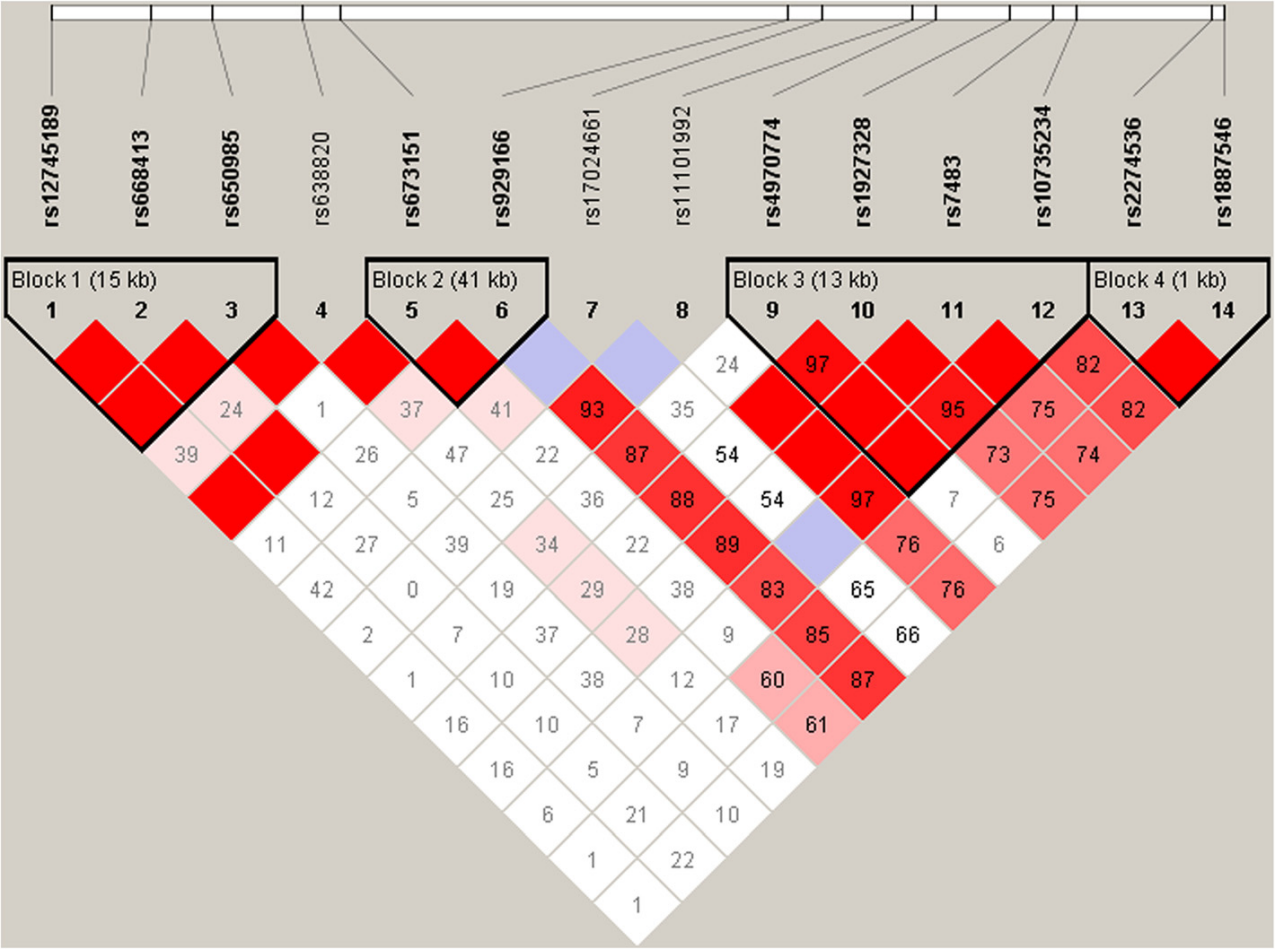
Linkage disequilibrium (LD) between SNPs in the region of the *GSTM* cluster in a combined Russian sample. A standard Haploview *D*′/LOD color scheme is used to demonstrate LD, with bright red for strong LD (LOD ≥ 2, *D*′ = 1), white for no LD (LOD < 2, *D*′ < 1), shades of pink/red for intermediate LD (LOD ≥ 2, *D*′ < 1), and blue for statistically ambiguous LD (LOD < 2, *D*′ = 1) [14]. Numbers in cells represent *D*′ values between pairs of SNPs (empty cells indicate that *D*′ = 1 between the corresponding SNPs). Black triangles indicate inferred haplotype blocks

Fourteen SNPs determined as located in the region of the *GSTM* cluster were found among SNPs from the Illumina chip analyses and used in the current study. Figure 1 shows the LD between the SNPs, and the haploblocks inferred in the combined Russian sample. In total, four haploblocks were inferred in the chromosome region. We started our analysis from haplotypes of haploblock 2, comprising SNPs rs673151 and rs929166. These SNPs were the nearest to the region of the *GSTM1* deletion. The frequencies of haplotypes CT and TT were maximal in the group of individuals with a *GSTM1 +/+* genotype, while the third haplotype, CG, was the most frequent among the carriers of the *GSTM1* −/− genotype. All three haplotypes had intermediate frequencies in the group of individuals with the *GSTM1 −/+* genotype (Table 2). The same picture of haplotype distribution (i.e., intermediate values of haplotype frequencies in the group of individuals with the *GSTM1* −/+ genotype) was observed for haploblocks 1, 3, and 4 (Table 2). As a consequence, pairwise comparisons of haplotypes showed the greatest differences between individuals with the *GSTM1* −/− genotype and individuals with the *GSTM1* +/+ genotype. Haplotype distributions in all four blocks were significantly different between these two groups (Table 3). A slightly lower number of significant differences were found between the groups with *GSTM1* −/− and *GSTM* −/+ genotypes, and only one when the group with the *GSTM1* +/+ genotype was compared with the group with the *GSTM* −/+ genotype. The same analysis was also carried out in the CEU population. The found correlations in haplotype distributions were similar to those observed in the Russian sample.

**Table 2:** Haplotype frequencies in groups of individuals subdivided according to *GSTM1* deletion polymorphism

Number of haploblock	Haplotypes	Individuals with *GSTM1* −/− genotype (2 N = 256)	Individuals with *GSTM1* −/+ genotype (2 N = 242)	Individuals with *GSTM1* +/+ genotype (2 N = 72)
1	TCT	0.390	0.512	0.597
	CAT	0.508	0.380	0.250
	CCT	0.028	0.069	0.125
	CCC	0.075	0.039	0.028
2	CT	0.583	0.705	0.736
	CG	0.406	0.237	0.139
	TT	0.012	0.055	0.125
3	AACG	0.390	0.392	0.319
	CGTA	0.374	0.254	0.167
	AACA	0.154	0.188	0.306
	CACA	0.079	0.158	0.208
	CGCA	0.004	0	0
	AGCG	0	0.008	0
4	GT	0.217	0.343	0.542
	AG	0.776	0.657	0.458
	GG	0.008	0	0

**Table 3:** Statistics (*P*-values) of intergroup comparisons of haplotype frequencies^a^

Number of haploblock	Individuals with *GSTM1* −/− genotype vs. Individuals with *GSTM1* +/+ genotype	Individuals with *GSTM1* −/− genotype vs. Individuals with *GSTM1* −/+ genotype	Individuals with *GSTM1* +/+ genotype vs. Individuals with *GSTM1* −/+ genotype
1	**‹0.0001**	0.0060	0.9512
2	**‹0.0001**	**‹0.0001**	0.0391
3	**‹0.0001**	**0.0038**	0.0700
4	**‹0.0001**	**0.0022**	**0.0024**

^a^significant differences are in bold

**Fig. 2:**
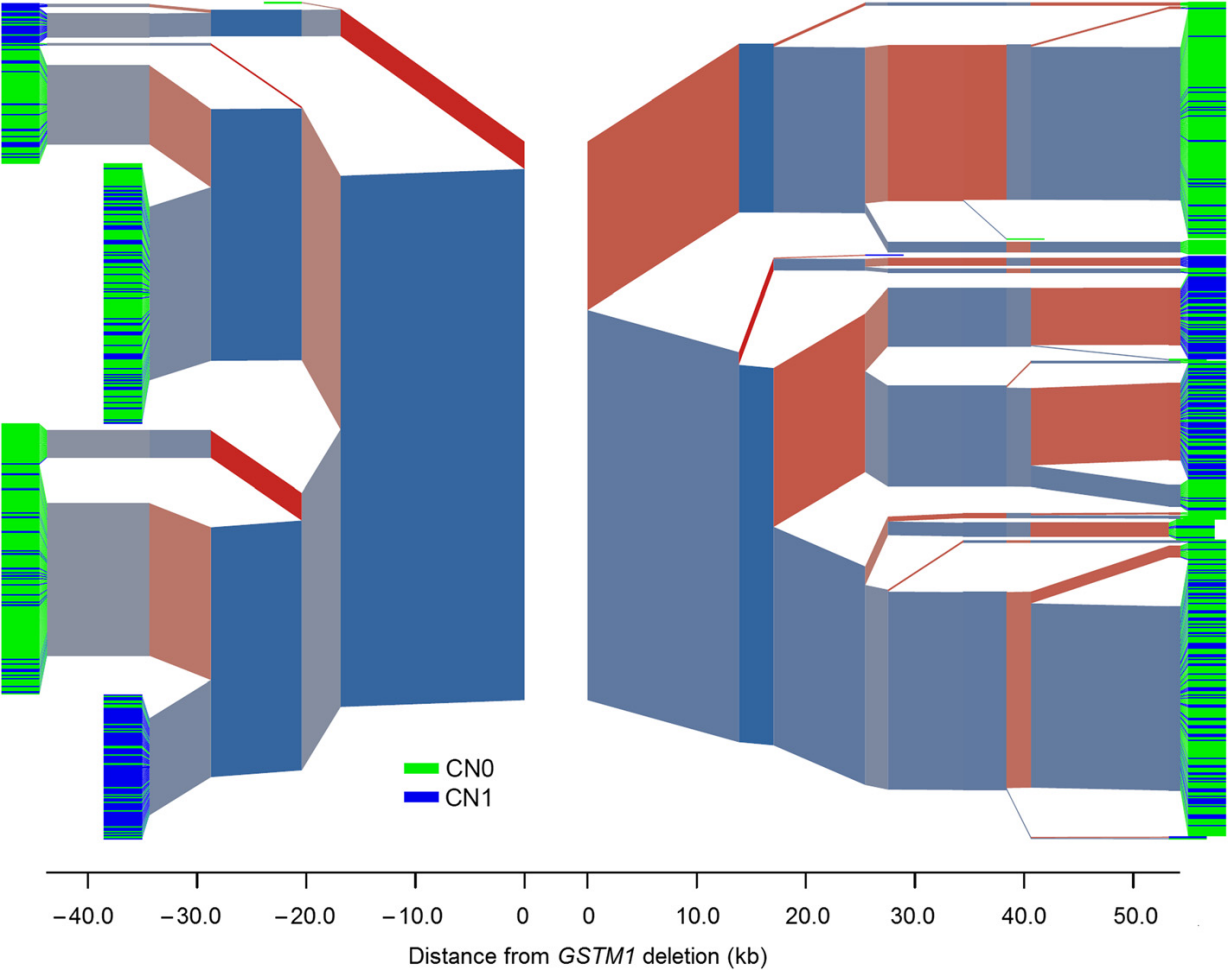
*GSTM1* copy number distribution and haplotype structure of the *GSTM* cluster genomic region in a combined Russian sample. SNP haplotypes in the region of the *GSTM* cluster are shown. The gap in the center of the plot indicates the edges of the *GSTM1* deletion. The branch points represent SNPs at which flanking haplotypes diverge because of mutation or recombination. The thickness of the branches corresponds to the frequency of haplotypes. Blue to red color intensity indicates the allele frequency of individual SNPs used to define the haplotypes, where the major allele is bluer, and the minor allele is redder. The color of the “leaves” at the ends of the branches indicates the *GSTM1* state of the chromosomes: green, no copies of *GSTM1* (deleted gene; CN0); blue, one functional copy of *GSTM1* (CN1)

**Fig. 3:**
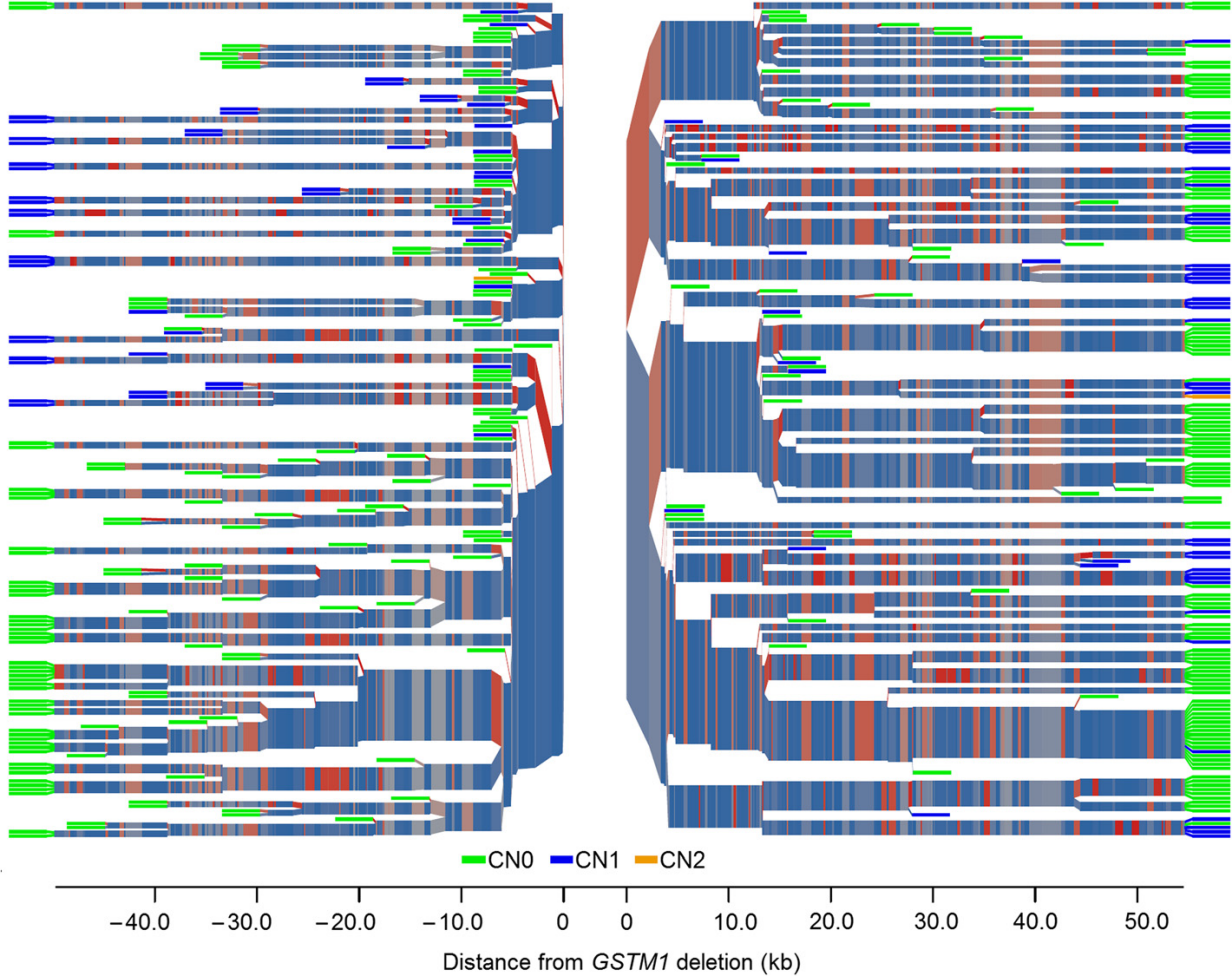
*GSTM1* copy number distribution and haplotype structure of the *GSTM* cluster genomic region in the CEU population. The plot has been generated based on the data on the phase state of the alleles of *GSTM1* and 356 SNPs (MAF ≥ 0.01). One individual had two copies of *GSTM1* on one of his chromosomes (CN2), which is indicated as an orange “leaf” in the right-hand part of the plot. Other designations are the same as in Fig. 2

The output of processing phased Russian genotypes generally supported the results of the haploblock-based analysis. The output was expressed as an unequal occurrence of *GSTM1* alleles on different haplotypes (Fig. 2). Understanding the paucity of the SNP set tested, we attempted to increase the resolution by imputing additional genotypes. However, the additional genotypes did not markedly influence the pattern of haplotypes inferred, although the total number of SNPs increased to 49. This might be because none of the new SNPs were closer to the *GSTM1* deletion than the two aforementioned SNPs, rs673151 and rs929166, which could be the result of earlier and crucial branching. The relevance of earlier branching was supported by the data from the processing of a set of 356 phased SNPs with a MAF ≥ 0.01 in the CEU sample, in which some SNPs were located at some hundreds of base pairs from the deletion (Fig. 3). Furthermore, in the resulting plot, nonrandom occurrence of particular *GSTM1* alleles at different haplotypes was more evident (i.e., the lower left part of the plot was occupied exclusively by haplotypes with a *GSTM1* null allele) (Fig. 3).

## Discussion

The *GSTM1* homozygous deletion is very common in human populations, varying between 16 and 40 % in Africans (sub-Saharan Africa), 42 and 55 % in Europeans, and 42 and 65 % in East Asian populations [3]. The frequency of the deletion suggests that *GSTM1* has not been subjected to strong environmental selection pressure during evolution, and thus it might have resulted from an ancestral deletion that was widely spread across populations because of a founder effect.

An alternative explanation can be proposed in the context of the molecular structure of the *GSTM1* region. Xu and coauthors [4] found that *GSTM1* in *GSTM1* +/+ individuals was flanked by two highly homologous 4.2 kb regions. By contrast, individuals with a completely deleted *GSTM1* had only one segment that was identical to the regions in *GSTM1* +/+ individuals. Such a structural organization of the region (i.e., existence of two highly homologous repeats) allows for the possibility of nonallelic homologous recombination in the chromosomal area, resulting in gene deletion [18], particularly the *GSTM1* deletion, which in principle might occur independently on multiple occasions. The results of our intergroup haplotype comparisons support the hypothesis that the deletion might occur at different haplotypes because there were no differences in haplotype spectra between the groups. At the same time, substantial differences in the frequencies of the haplotypes were found. The greatest differences were observed between groups of individuals with homozygous *GSTM1* −/− and +/+ genotypes, which differed in haplotype frequency in all four haploblocks inferred. We hypothesized that the differences may show the potential influence of genomic context on the occurrence of the deletion. The assumption was supported by the results of exploring structural haplotypes in the region of the *GSTM1* deletion, where unequal occurrence of *GSTM1* − and + alleles at different SNP haplotypes was demonstrated. In what way could the context affect the deletion frequency (appearance)? It is unlikely that individual SNPs could affect recombination if they were not in a recombination hotspot [19]. However, taking into account the data on the possibility of functional compensation of *GSTM1* −/− by *GSTM2* [8], one can suggest that the correlations found may reflect the existence of functional associations between *GSTM1* and allelic variants of other genes of the *GSTM* cluster. Such associations can explain both why *GSTM1* has not been subjected to strong environmental selection and the high frequency of *GSTM1* deletion. The associations seem to be also relevant to the conflicting results reported in studies that correlated *GSTM1* with risk of cancer and other diseases [6, 20–24] in which the absence of GSTM1-related enzymatic activity could be masked by catalytic activities of other GSTMs and resulted in no or reduced impact of *GSTM1* deletion on disease risk. Finally, taken in the context of the results of associative studies, our findings highlight the necessity of parallel examination of allelic status of functionally and structurally related members of the gene family.

## Conclusions

In summary, we have reported the results of exploring the haplotype structure in the *GSTM* cluster region in relation to *GSTM1* deletion polymorphism. By using both haploblock-based and extended phased haplotypes, substantial differences in haplotype distribution were observed when they were correlated with the *GSTM1* genotypes and alleles. The results suggest the potential role of genetic context in *GSTM1* deletion frequency (appearance), and in the determination of deletion-related effects.
